# Toward a roadmap for sustainable lean adoption in hospitals: a Delphi study

**DOI:** 10.1186/s12913-024-11529-4

**Published:** 2024-09-18

**Authors:** Maria M. Van Zyl-Cillié, Desirée H. van Dun, Hanneke Meijer

**Affiliations:** 1 Faculty of Engineering, North-West University, 11 Hoffman Street, Potchefstroom, South Africa; 2 Faculty of Behavioural, Management and Social Sciences, University of Twente, Drienerlolaan 5, Enschede, 7522 NB The Netherlands

**Keywords:** Lean in healthcare, Lean implementation, Change management, Maturity models, Implementation science

## Abstract

**Background:**

The benefits of lean adoption in healthcare include improved process efficiency and quality of patient care. However, research indicates that lean implementation in healthcare, and specifically hospitals, is often not sustained. Furthermore, there is a need for maturity models that guide lean implementation, specifically in hospitals. This study develops a prescriptive maturity model named the Sustaining of Lean Adoption in Hospitals Roadmap (SOLAR) that acts as a practical guideline for the sustainable adoption of lean in hospitals.

**Methods:**

The SOLAR has three theoretical foundations, namely lean implementation success factors in hospitals, implementation science, and change management theory. A systematic literature review was conducted to determine the lean implementation success factors in hospitals as the first building block. Secondly, practices from implementation science were used to create the action items in the SOLAR. Ten change steps were elicited from change management theory as the third theoretical building block of the roadmap. We refined the roadmap through three Delphi rounds that verified its useability in hospitals.

**Results:**

The final SOLAR consists of four maturity phases (prepare, plan, experiment and learn, and sustain) and includes action items for each phase related to the hospital’s strategy, resources, engaging of people, and culture. The action items and change management steps shown in the SOLAR are *not* intended as an exhaustive list but provide guidelines on aspects hospitals must consider when they aim to adopt lean sustainably.

**Conclusions:**

The strong theoretical base of the SOLAR enables hospitals to safely experiment and learn which implementation methods are best suited to their unique environment. The SOLAR is, therefore, an actionable guideline that informs both academics and practitioners involved in lean adoption in hospitals. This roadmap can guide future retrospective longitudinal or action research.

**Supplementary Information:**

The online version contains supplementary material available at 10.1186/s12913-024-11529-4.

## Background

Hospitals, also called inpatient care services, experience many operational challenges. Delivering healthcare services efficiently whilst improving the flow and reducing patients’ waiting time is one of these challenges [1]. Lean management, which originated in the manufacturing industry, has proven to drive improved efficiencies in the healthcare sector in general and in hospitals in particular [2] as well as improved quality of patient care and overall performance gains [3]. Many hospitals have implemented lean in recent years due to these benefits. Lean implementation requires a complete change in organisational culture and thinking, but adopting lean tools does not ensure that the implementation is sustainable or has been adopted as part of the organisation’s culture. This is confirmed by several researchers, like Van Rossum et al. [4] who argued that lean implementation in healthcare organisations is not always maintained. In the healthcare environment, lean adoption is only seen as successful if the implementation thereof *permanently* improves the quality of service and patient satisfaction [5]. Van Beers et al. [3] further argued that lean implementation in hospitals often does not achieve the desired results and is a lengthy process. Indeed, Akugizibwe and Clegg [6], observed that healthcare providers (such as hospitals) struggle to sustain the success achieved after initial lean implementation.

Implementing continuous improvement interventions such as lean, Total Quality Management and Six Sigma, is often challenging due to the organisational change management process it requires [7]. In addition, such implementations are complicated due to, amongst other things, the complexity of healthcare organisations [8–10]. These complexities include the typical organisational structures of hospitals where different units often function in isolation as their own profit and loss entities, with little motivation for functioning across silos. In addition, hospitals have strict hierarchical structures [11] and not all stakeholders involved in a patient’s journey (such as physicians) are employed by the hospital, making it difficult to ensure that they buy into the hospital’s lean journey.

Models and frameworks that guide the successful implementation of continuous improvement initiatives in organisations do exist. Despite the availability of such maturity or implementation models [12], continuous improvement implementation initiatives have a high failure rate [7]. In line with this, researchers contend that there is minimal evidence of lean healthcare implementations sustained over the long term [13]. Indeed, D’Andreamatteo et al. [14] found that although the factors that contribute toward successful lean implementation in healthcare are established in the literature, research on adopting lean sustainably and the implementation process of lean in healthcare is lacking. Henrique et al. [15] made a first attempt to aggregate key factors that might influence the sustainability of lean interventions in hospitals. Furthermore, Kunnen et al. [16] thematically analysed the barriers and facilitators that influence the sustainable adoption of lean in healthcare organisations, but not specifically in hospitals.

Lameijer et al. [7] found that while implementation readiness factors often form part of implementation guidelines or maturity models, factors related to the sustainability of results are lacking. Furthermore, the available guidelines do not address contextual factors such as the industry or environment. Indeed, Andersen et al. [17] emphasise the importance of tailoring lean specifically for hospitals. Similarly, Antony et al. [18], Zanon et al. [19], and our own literature review identified the lack of a fully developed framework and assessment methodology for lean implementation, specifically at the hospital level. In addition, although prescriptive maturity models can provide organisations with the general direction for deploying lean, they do not necessarily guide implementation using clear action items [12]. Lameijer et al. [7] argued that there is thus a need for industry- and implementation-specific guidelines or maturity models to boost the success and durability of lean initiatives.

In sum, although lean can address prominent challenges in hospitals there is a gap in the literature on how to sustain lean in hospitals [14]. With many hospitals facing pressure to improve their financial performance, efficiency and patient care quality, there is a critical need for guidelines on sustaining lean in such settings. This research aims to design a prescriptive maturity model, the *S*ustaining *o*f *L*ean *A*doption in Hospitals *R*oadmap (SOLAR), that will help guide practitioners and scholars alike towards sustainable lean implementation in a hospital environment. The first research phase entailed developing the SOLAR from solid theoretical principles: The known success factors for lean implementation, change management theory, and the relatively novel theory of implementation science. In the second research phase, the proposed model was tested utilising a three-round Delphi study, during which feedback from lean healthcare expert practitioners and academics was obtained.

The resulting roadmap is intended to guide the lean adoption process in hospitals through action items throughout the change management process. Furthermore, the SOLAR contributes to the literature by integrating known lean implementation success factors and change management theory with implementation science. The resulting multidisciplinary model takes various prominent features of the hospital setting into account, including the risk-aversity of hospital staff members and the hierarchical, siloed organisational structure, requiring many stakeholders’ involvement beyond only identifying customer/patient value.

The next section provides an overview of the theory on which the initial SOLAR is built. The methodology section explains how the SOLAR was developed in dialogue with experts across the globe. The results section then discusses the content of the SOLAR, after which the theoretical and practical implications are drawn in the final discussion section.

## Initial SOLAR development: literature review

The first phase of developing the SOLAR was to establish the building blocks from the literature. A brief background to the purpose and use of maturity models is provided, after which lean implementation success factors, implementation science, and change management theory are reviewed.

### Maturity models

Becker et al. [20] summarised a maturity model as a guide to organisational transformation from an initial to a desired state, where the model offers the maturity levels to guide organisational transformation. Maturity models are generally applied for two reasons. Firstly, to determine the current maturity level of an organisation [21]. Maturity models in this context are called descriptive maturity models [22] and are used to assess an organisation’s progress to achieve a desired level of maturity. Secondly, to guide the organisation’s journey to the desired state, i.e. prescriptive maturity models [21] that typically include detailed actions developed from historical data to prescribe organisational transformation [22].

Maturity models can be used in lean deployment to guide organisations on what steps to take to achieve sustainable lean adoption *or* to assist organisations in assessing how far along the journey towards complete lean adoption they are. Yet, lean adoption is a long-term venture, and many argue that it has no clear ‘end’ because it aims for continuous improvement. Some authors refer to the level at which an organisation has adopted lean as ‘leanness’, i.e. the extent to which lean practices have been adopted and the resulting performance achievements [19]. Ways to assess the extent to which lean has been infused into an organisation, include benchmarking [23], storytelling [24] and assessment tools such as the ‘Lean Enterprise Self-Assessment’ [25]. Maturity models can also act as evaluation tools to determine an organisation’s current state and guide toward achieving a desired state [26].

A review by Zanon et al. [19] revealed 19 lean maturity models that are presented in the literature. All models assess the general adoption of lean in “phases” or “milestones”, both of which are synonymous with “maturity levels”, and the extent to which maturity has been achieved is measured against different criteria. These 19 maturity models are predominantly descriptive. In order to determine the maturity levels of the SOLAR, we investigated the terms used in the models presented by Zanon et al. [19] and two models [22, 23] from our own review of lean maturity models. The six lean maturity models with their respective descriptions of maturity phases are summarised in Table 1. It was found that all of the models described progressive phases with unique, diverse labels. The phases of maturity are described in intervals of between four and eight steps.

**Table 1:** Maturity phases of descriptive maturity models

No.	Reference	Maturity Phases
1	Tortorella, Vergara, and Ferreira [27]	Rating of maturity according to a 5-point scale. 1 = Practice has not been implemented 5 = Full implementation of the practice
2	Jørgensen et al. [28]	1. Sporadic production optimisation 2. Basic lean implementation 3. Strategic lean interventions 4. Proactive lean culture 5. Lean in the extended manufacturing enterprise
3	Tortorella et al. [29]; Tortorella and Fogliatto [30]	1. Adopt lean paradigm 2. Prepare implementation 3. Define value 4. Identify flow of value 5. Design production system 6. Implement flow 7. Implement pull system 8. Look for perfection
4	Marsilio et al. [23]	1. Still in the new start-up stage 2. Beyond start-up, but challenged moving forward 3. Expanding to other units and getting traction 4. Mature transformational performance improvement
5	Maier et al. [22]	1. Planning 2. Development 3. Evaluation 4. Maintenance
6	Verrier et al. [31]	1. Initial stage (limited awareness) 2. Managed stage (occasional use of practices) 3. Defined stage (regular separate conduct of practices) 4. Quantitatively managed staged (regular combined practice conduct 5. Optimisation (continuous improvement through lean)

Because of this diversity, Zanon et al. [19] proposed that lean maturity levels be described as follows:

1. Level 1 is associated with some (small) lean initiatives being undertaken, which are not fully integrated into the organisation. This level description is similar to, amongst others, level 1 (initial stage, limited awareness) of Verrier et al. [31] as well as level 1 (adopt lean paradigm) presented by Tortorella et al. [29]. During this level, preparation for adopting lean in the organisation, typically occurs.
2. Level 2 is the phase during which customer value is identified and improvements and lean implementation are directed towards isolated areas in the organisation [19]. This level corresponds to levels 2 and 3 (basic lean implementation and strategic lean implementation) of Jørgensen et al. [28] as well as levels 3 and 4 of Tortorella et al. [29] (define value and identify flow of value). In essence, this phase focuses on planning the lean adoption of the organisation and how the lean adoption will realise value.
3. Level 3 is described by Zanon et al. [19] as the phase during which improvement initiatives are aligned, and stakeholders can observe how process improvements contribute towards performance metrics. This description is similar to level 4 (quantitatively managed stage) of Verrier et al. [31], level 3 of Marsilio et al. [23] (expanding to other units and getting traction) as well as level 4 (proactive lean culture) as presented in the work of Jørgensen et al. [28].
4. The final level of lean maturity is characterised by the continuous use of lean concepts throughout the organisation and focuses on sustaining lean adoption in the organisation [19]. Verrier et al. [31] describe this level as optimisation (continuous improvement through lean). Marsilio et al. [23] refer to this level of maturity as “mature transformational performance improvement” and Maier et al. [22] as “maintenance”.

Furthermore, maturity levels are typically associated with capabilities and activities that an organisation needs to perform or are measured against as they progress on a maturity path [32]. While investigating such progress of improvement, Netland and Ferdows [33] observed that an S-shaped operational performance improvement occurs in phases over time. During the initial phases of lean implementation, operational improvement occurs slowly, followed by a drastic and rapid improvement, whereafter the improvement gradually tapers off [33]. This non-linearity of business performance improvement during lean adoption was confirmed by Negrão et al. [34]. At the saturation point lean adoption is mature and can be sustained if the correct focus is maintained.

**Fig. 1:**
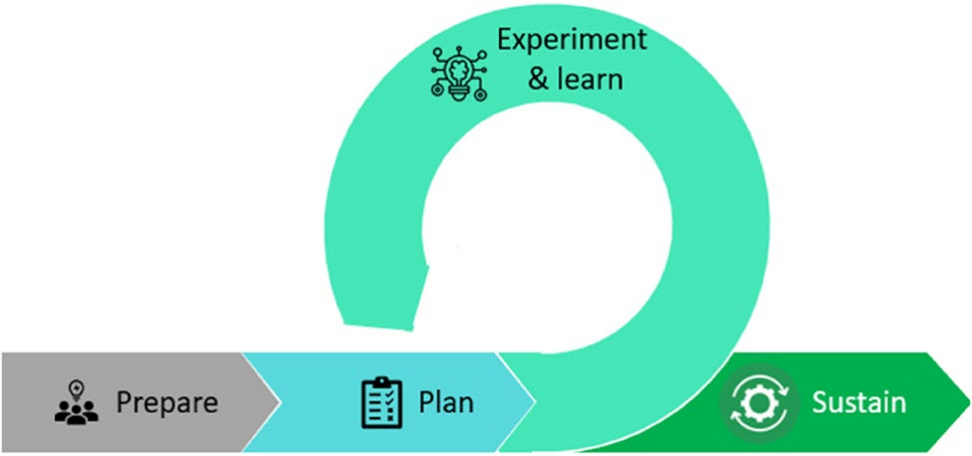
Sustainable lean hospital adoption roadmap maturity phases

In sum, in keeping with the notion that lean maturity is achieved in phases whereby there must be room for continuous improvement to sustain lean adoption over time, we developed our SOLAR as a prescriptive maturity model comprising four phases deduced from our overview of lean maturity models, as shown in Fig. 1: Prepare, Plan, Experiment and Learn, and Sustain.

### Lean implementation success factors

The second building block of the SOLAR is informed by literature-based factors that could influence the successful adoption of lean in a hospital environment. These factors, amongst others, are described as *barriers*, *facilitators*, *challenges*, *readiness factors*, *success factors*, *inhibitors*, and *managerial attributes* [35–40]. We refer to them as success factors for brevity. In terms of lean deployment, success factors are those that enable employees to adopt lean thinking in their everyday routines [41] and can be seen as part of a change-implementation strategy that influences the sustainability of the change [8]. It is, therefore, critical to incorporate success factors into a lean healthcare adoption maturity model.

This research follows a similar approach to that of Kunnen et al. [16] but is specific to a hospital environment. Hence, a systematic literature review (SLR) was conducted at the start of this study in 2019 to determine the success factors necessary for lean implementation and adoption in hospitals, and integrated into the SOLAR by addressing the following research question: *What factors influence lean implementation success within a hospital environment?*

In conducting the SLR following the PRISMA statement [42], nineteen articles on lean implementation success factors were selected following the systematic approach proposed by Siddaway et al. [43]. The search terms used in the search databases Scopus and EBSCOhost (which included databases such as Academic Search Premier and MEDLINE) were as follows:

(“lean” OR “continu* improvement”) AND (“implement” OR “deploy*” OR “adopt” OR “adapt” OR “appl*” OR “conscious*” OR “integrat*”) AND (“health care” OR “healthcare” OR “hospital” OR “clinic” OR “health cent*” OR “medical service” OR “medical care environment” OR “medical facility*” OR “medical cent*”) AND (“success factor*” OR “success” OR “critical factor*” OR “change factor*” OR “driver” OR “important factor” OR “facilitate*” OR “sustain” OR “long term” OR “long term” OR “read* factor*” OR “failure factor*” OR “challenge” OR “barrier” OR “lesson*” OR “issue”).

As inclusion criteria, only English papers with available full texts, published in accredited journals or established (peer-reviewed) conference proceedings, and focused on one or more factors influencing lean implementation in a hospital setting were selected by one author (HM), and then independently checked by the first author (MVZ-C). These inclusion criteria meant to account for the relevance and quality of the included papers. Book chapters and studies executed outside of a hospital environment, in non-service parts of the hospital, or those concerned with implementing lean in combination with another methodology, such as Six Sigma, were excluded. In particular, studies combined with Six Sigma were excluded due to their specific focus on quantitative statistical process control initiatives and not primarily on lean success factors. The final selection of papers was then determined by the entire author team (including also DVD); to avoid any omissions, the papers were discussed elaborately.

**Fig. 2:**
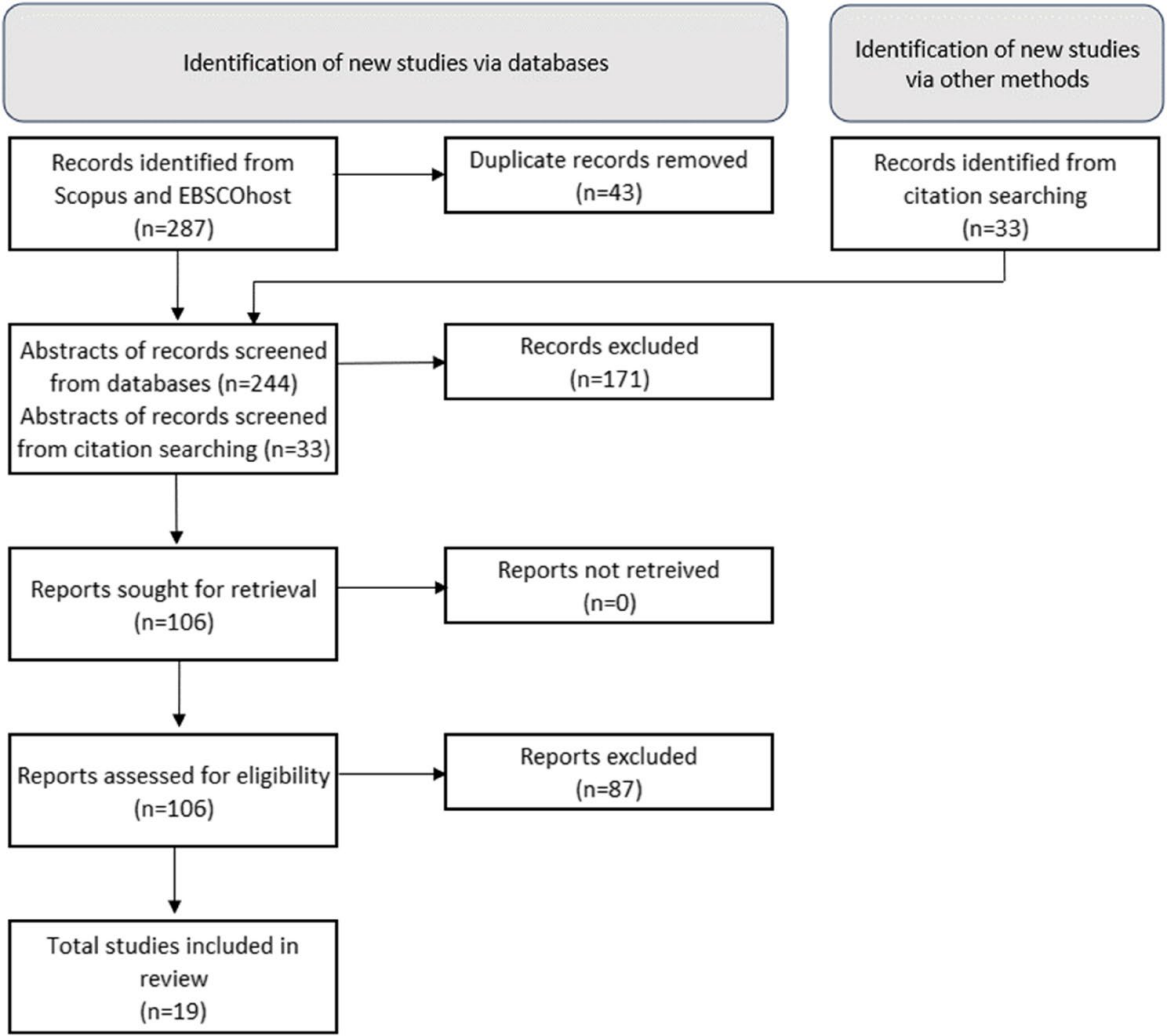
Systematic literature review approach to determine lean adoption success factors, following the PRISMA statement

Before analysing the selected studies in more depth, the author team screened the journal impact factors as well as methods used and rigour to account for the quality of the corpus. SCImago Journal Ranking indicator, which assesses the impact and influence of journals independently, was consulted, and we found that 12 of the 19 articles in our sample were published in the top 25-50% (quartiles one and two) journals. Four articles were published in quartile three (top 75%) journals, two in peer-reviewed conference proceedings, and one in a quartile four journal. The journal ‘Quality Management in Health Care’ (quartile two journal) contributed the most articles (3 articles). The methods followed in our sample ranged from semi-structured interviews (7 articles), literature reviews (6 articles), field observations (2 articles), and quantitative methods such as structural equation modelling (4 articles). The diversity of the sample of selected papers, both in terms of methodology and countries of data collection ranging from Sweden to Iran, is proposed to curb any remaining biases in the selected studies, allowing for high-quality insights. The SLR approach, following the PRISMA statement, is summarized in Fig. 2.

In terms of content analysis, any mention of factors influencing the success of lean implementation within a healthcare environment was extracted from the selected studies. To minimize bias and ensure that all relevant factors were collected, we followed the 21-item ENTREQ guidelines [44]. Firstly, one author (HM) extracted factors influencing successful lean implementation from the selected studies. Then, a second author (MVZ-C) reviewed the selection of factors and compared them to the nineteen selected studies to ensure a balanced view. In line with Kunnen et al. [16], inductive reasoning was used to create labels for similar factors. The two authors further refined the factor labels with the third author (DVD) whereafter the factors were grouped under four themes: (1) strategy, (2) resources, (3) engaging people, and (4) organisational culture. Table 2 depicts each theme, corresponding lean adoption success factors, and the original sources which identified them.

**Table 2:** Lean adoption success factors identified in the 19 selected articles

Factor	Source(s)
**Theme: Strategy**	
Define strategy	[14, 35, 37–39, 45–52]
Define value	[37, 38, 45, 47, 48, 52]
Implementation timeline	[39, 45, 47, 50]
Implementation process	[14, 38, 39, 46, 47, 49, 50, 52, 53]
Contextualisation	[46, 47, 49, 54, 55]
Management commitment	[14, 35, 37, 38, 46–50, 53, 54, 56]
Follow-up	[14, 36, 38, 39, 41, 45–48, 52]
**Theme: Resources**	
Financial resources	[39, 45, 47, 56]
Supporting resources	[3, 39, 52, 56]
Expertise	[14, 39, 49, 52, 56]
Healthcare staff structure	[36, 39, 46, 47, 53, 54]
Data collection	[36]
Process variability	[14, 37]
Defining waste	[14, 37]
Implementation team	[38, 46, 47, 49, 56]
**Theme: Engaging People**	
Patient engagement	[45, 47, 56]
Employee engagement	[38, 39, 45, 47, 49, 50, 52, 56]
Physician engagement	[39, 54, 56]
Management engagement	[14, 39, 41, 46, 50, 52]
Employee training	[14, 35, 37–39, 41, 46–49, 52, 54]
Lean philosophy	[14, 37, 45, 47]
Facilitator training	[38, 39, 46, 47, 52, 56]
General communication	[39, 46, 47, 50, 52]
Interdepartmental cooperation	[35–37, 39, 45, 47, 54]
Progress communication	[35, 45, 47]
Terminology	[36]
**Theme: Organisational Culture**	
Supportive culture	[14, 39, 45–49, 56]
Readiness and resistance	[14, 35, 36, 45, 47, 49, 50, 52, 56]
Normalisation of initiative	[36, 39, 46–48, 52–54, 56]
Learn from mistakes	[14, 56]
Organisational momentum	[35, 36]
Leadership style	[35, 39–41]

The success factors listed in Table 2 were used in conjunction with a well-researched framework from implementation science, as discussed in the next section, to develop the proposed action items of the SOLAR under each maturity phase.

### Implementation science

Implementation science, an emerging field in healthcare evidence-based standard practices adoption, was used as the theory that informs the third element of the SOLAR. Implementation science is concerned with the study of methods that aim to diffuse research findings and evidence-based practices into an organisation’s routine [57]. May and Finch [58] further defined implementation as a deliberate effort to introduce something new to an environment to bring about change.

According to the theory of implementation science, this change is realised in organisations through a diffusion-dissemination-implementation continuum [55], which implies an ever-evolving change process. This diffusion-dissemination-implementation continuum is valuable to improving the spread of research findings that could improve a healthcare environment [59]. Diffusion is the inactive part of imparting knowledge about new practices [55], whereas dissemination requires more action and actively communicating new practices to the target group to ‘helping it happen’ [59, 60]. Implementation is the deliberate action of ensuring that research findings are truly incorporated into the environment’s everyday practices [55]; in other words, ‘making it happen’ [60].

A key framework in the field of implementation science that guides the diffusion-dissemination-implementation process is the Quality Implementation Framework (QIF) [59]. This framework is suitable for informing the action items included in the SOLAR because the QIF may be generalised for any environment, it provides clear process steps for its application, and is widely cited and frequently used.

The QIF lists 14 critical steps in a four-phased approach that contributes towards a quality implementation where fidelity of the innovation is maintained throughout the implementation process [61]. Examples of these critical steps are determining the organisation’s current state regarding needs and resources, creating implementation teams, ensuring a supportive feedback system, and learning from the experience of implementing the change. Furthermore, the framework provides questions under each critical step the researcher needs to consider when implementing a change intervention. The proposed action items in the SOLAR were thus further developed by incorporating the QIF and its 14 critical steps.

### Change management theory

In organisational behaviour literature, it is contended that planned organisational change is more likely to succeed if the change process considers all organisational stakeholders, whereby change needs to occur in a group where individuals’ behaviour and reaction to change is a function of the group environment [62]. The theory of change management uses frameworks and mechanisms to manage change in an organisation whilst causing minimal negative disruption to the workforce [63].

Although many useful change management methods and theories have been developed, the variability in each organisation and change environment may require adjustment according to their specific context [64, 65]. Al-Haddad and Kotnour [62] explained the taxonomy of change in literature as consisting of change types, methods, enablers, and outcomes. The change type is classified in terms of the scale and duration of the change. Once the change type is defined, the most appropriate change method can be determined; these methods, in turn, are divided between systematic change methods and change management methods. Systematic change methods include processes and tools that assist organisational change agents (such as managers) to take change-related decisions [62]. These systematic change methods are cyclical and integrative, as opposed to some traditional change theories that mainly suggest management-driven change through incremental process adjustment. Examples of systematic change methods include Six Sigma, Total Quality Management and process re-engineering. On the other hand, change management methods are more conceptual and broader [62], as they assist management in aligning the change initiative with the overall organisational strategy and mission and embed the change into the organisational culture.

Al-Haddad and Kotnour [62] further argued that certain factors increase the probability of successful change and are known as organisational change enablers. Some examples of such enablers include setting a shared vision and direction for the change, clearly communicating the benefit and clarifying the roles of the employees involved in the change [63]. Notably, training employees and measuring the evolution of organisational change will also increase the probability of sustainable change [66]. Change outcomes, as depicted by Al-Haddad and Kotnour’s [62], relate to measuring the change’s performance from both a customer and organisational perspective. Errida and Lotfi [67] emphasise the importance of setting goals for such performance measures that are continuously tracked.

Furthermore, Stouten et al. [64] highlighted seven prescriptive change management models. These models (see Table 3) guide the management team through sequential steps in executing change interventions in their organisations. Some of the models corresponded with both the change management methods and systematic change methods [62]. Although lean implementation in a hospital environment will evolve organically and iteratively, it must be embedded in the hospital culture [63] which tends to be a large change stretched over an extended period. Therefore, change management methods [62] would be appropriate to guide lean implementation in hospitals, especially the prescriptive ones which provide specific guidance on steps to take. Hence, we focused on the prescriptive change management models classified by Stouten et al. [64]. In selecting the appropriate models to inform the SOLAR, those prescriptive change management models were filtered to ensure that they were also classified as change management methods by Al-Haddad and Kotnour [62]. Table 2 shows the result of the filtering process and the subsequent four change management models that are used to inform the SOLAR: (i) Lewin’s three-phase process method, (ii) Judson’s five steps, (iii) Kanter, Stein and Jick’s ten commandments, and (iv) Kotter’s eight-step model.

**Table 3:** Change management model classification

Change Management Model	Classified as Change Management Method (Al-Haddad & Kotnour [62])	Classified as Prescriptive Change Management Model (Stouten et al. [64])
Lewin’s Three-Phase Process Method	X	X
Judson’s Method	X	X
Beer’s Six-Step Change Management Model		X
Appreciative Inquiry		X
Kanter, Stein and Jick’s Ten Commandments	X	X
Hiatt’s ADKAR Model		X
Kotter’s Eight-Step Model	X	X

Stouten et al. [64] argued that many of the prescriptive models have similar practices and processes. The models also have a flow that acknowledges the start of the change intervention followed by the dissemination and, finally implementation or adoption of the change. As such, Stouten et al. [64] synthesised these prescriptive change management models into ten change steps, starting with assessing the opportunity to motivate the change and ending with institutionalising the change in the organisational culture and practices. Given the overlap with Al-Haddad and Kotnour [62], we contend these ten change steps are a comprehensive synthesis of prescriptive change management models and change management methods included in this SOLAR.

## Methods

### Research design

Given the exploratory aim of the research, a Delphi study was conducted where the initial literature-inspired design of the prescriptive maturity model was refined through feedback from lean healthcare experts. The Delphi method elicits the opinion of a panel of experts over multiple rounds on a specific research subject [68, 69]. Expert feedback was collected from two rounds of online surveys and from narrative interviews in the third and final round, whereby the initial model was amended after each round. The surveys and the questions used in the narrative interviews were designed based on the approach followed by Tortorella et al. [70] and further refined after several dry-runs among the author team. They can be found in Additional File 1. The result of the Delphi study is the model we named ‘SOLAR’, presented herein.

### Sampling approach and sample description

Delphi study respondents were selected to complete the first-round survey based on their knowledge and experience in implementing lean in hospital environments and their availability and willingness to participate [71, 72]. A purposive expert sampling technique was followed, complemented by snowball sampling to avoid selection bias [73]. Thus, members from the Southern African Industrial Engineering (SAIIE) society were contacted via e-mail. Respondents with experience in academia, public healthcare, and private healthcare were thus identified to form a heterogeneous lean expert group. The respondents were requested to forward the survey to other potential respondents who met the inclusion criteria thereby completing the snowball sampling process. For the second Delphi round, the same method was followed and the recruitment list was expanded to include lean healthcare experts from the Netherlands. Since the third Delphi round was used to validate the SOLAR, respondents from South Africa and the Netherlands who participated in the second round were selected to participate in this final round.

During the first round, 14 participants responded to the online survey. Their experience was balanced between private and public healthcare and academia. The majority of respondents (10 out of 14) were male and six of the respondents had more than 10 years of experience. The second round also elicited responses from 14 individuals, five of whom also participated in the first round. Most respondents of this second Delphi round indicated their lean in healthcare experience as private healthcare, nine were male and five female. All four respondents (three males, one female) who participated in the third round also participated in the second round, and one of them also took part in the first round. The respondents’ experience in lean in healthcare was equally represented by public and private healthcare as well as academia. Table 4 summarises the respondent data for all three Delphi rounds.

**Table 4:** Respondent Data for the three Delphi rounds

	Round 1			Round 2			Round 3	
Area of Expertise	Nr.	%		Nr.	%		Nr.	%
Academic	0	0%		2	14.3%		1	25.0%
Private Healthcare	3	21.4%		6	42.8%		1	25.0%
Public Healthcare	2	14.3%		4	28.5%		0	0%
Academic and Private Healthcare	1	7.1%		0	0%		0	0%
Academic and Public Healthcare	3	21.4%		0	0%		0	0%
Private and Public Healthcare	2	14.3%		0	0%		0	0%
Academic, Private and Public Healthcare	3	21.4%		2	14.3%		2	50.0%
**Total**	**14**			**14**			**4**	
**Years of Lean Healthcare Experience**								
0 to 2 years	2	14.3%		1	7.1%		0	0%
2 to 5 years	2	14.3%		4	28.5%		2	50.0%
5 to 10 years	4	28.5%		5	35.7%		1	25.0%
> 10 years	6	42.8%		4	28.5%		1	25.0%

### Data collection

#### Delphi round 1 – approach and outcomes

The initial prescriptive maturity model was presented to respondents in an explanatory video, followed by an online survey (Supplementary Table 1, Additional file 1) which consisted of multiple closed-ended questions. Specifically, respondents were asked to indicate to what degree they agreed with the statement: ‘*Although initial lean implementations in hospitals might be successful*,* it is often not sustained’* and: *‘The maturity model contributes towards the sustainability of lean implementation in a hospital’*. Respondents rated their level of agreement on a five-point scale ranging from ‘strongly disagree’, ‘disagree’, ‘undecided’, ‘agree’, or ‘strongly agree’. The survey also contained an open field for suggestions for improvement of the maturity model.

Ten out of 14 respondents agreed that hospitals often do not sustain lean implementation. Although 11 of the 14 respondents agreed that the initial maturity model contributed towards lean sustainability in hospitals, suggestions for improvement were also made. One respondent noted that the original naming of the four maturity phases (i.e., prepare, plan, implement and sustain) did indicate a clear implementation path but did not indicate how maturity evolved. Another respondent argued that the lean implementation strategy needs to be aligned with the hospital’s strategy. Another point of feedback was that the model’s action items should be more descriptive to be more actionable. Based on this feedback the model was altered incorporating change management theory, renaming the maturity phases, and refining the action items to be more descriptive and aligned with respondents’ feedback.

#### Delphi round 2 – approach and outcomes

The amended model was presented to respondents in a second Delphi round, using the same method as round one. The survey questions for the second round can be found in Supplementary Table 2, Additional file 1. Although some questions were similar to the first round, to evaluate the model’s usefulness, new questions were posed, such as ‘*Do you agree that the action items of the maturity model address all the relevant steps that need to be taken to successfully implement and sustain Lean in a hospital?’*

The results from this round indicated that seven out of 14 respondents agreed that lean implementation in hospitals is often not sustained. Twelve respondents agreed that, once the four phases of the maturity model and the corresponding action items were completed, lean implementation in a hospital would be sustained over the long term. Furthermore, ten respondents indicated that the model could be applied to any hospital setting. Some suggested changes regarding how the change steps were integrated within each model phase whereas others noted that actions within lean implementation were *‘ongoing*,* iterative*,* and circular’*. Respondents also commented that it was a *‘very elaborate and well thought through model’* and *‘I can see that a well-structured*,* scientific method was followed’.* The feedback from this second round helped alter the model to clarify how change steps were associated with maturity levels and to rename the third maturity level to “Experiment and Learn”. Action items were further refined.

#### Delphi round 3 – approach and outcomes

During the one-on-one online interviews of the third round, the final prescriptive maturity model was shared with the four respondents who took part in the second round and offered differing viewpoints. During these interviews, the researcher(s) presented the final SOLAR and the revisions based on the second round. (Supplementary Table 3, Additional File 1). The first question we asked was ‘*Do you agree with the naming of the model?*’. We also asked whether *‘the presentation of the phases of the maturity model was clearer’*. These questions stimulated an open conversation. The narrative that followed generally indicated that respondents were now clear that the aim of the prescriptive maturity model was to act as a guideline rather than a set of instructions. All respondents agreed that the final SOLAR was sound. Respondents also supported naming the third phase as ‘experiment and learn’, saying that *‘it’s very clear now that it’s cyclical’.* Regarding the model’s usefulness, respondents said they *‘really thought this made sense from a theoretical and practical standpoint’* and *‘it is a useful model and the updates are practical’*. The final SOLAR, the result of a thorough theoretical investigation and three Delphi rounds, is presented in the next section.

## Results

The final SOLAR is a prescriptive maturity model consisting of four phases: Prepare, Plan, Experiment and Learn, and Sustain. The underlying action items are informed by lean implementation success factors, as discussed in Sect. 2.2, and by the 14 critical steps of the QIF discussed in Sect. 2.3. The action items of each phase are presented under four themes, namely strategy, resources, engaging people, and culture. The final element of the SOLAR is change management theory: The ten change steps, derived from Stouten et al. [64] are highlighted and incorporated during each phase and theme of the SOLAR. The action items and change management steps shown in the SOLAR are *not* intended as an exhaustive list but provide guidelines on aspects one must consider for a hospital that aims to adopt lean sustainably. Table 5 depicts the final SOLAR, which is discussed here in relation to the literature.

**Table 5:** Sustaining of lean adoption in hospitals Roadmap (SOLAR)

Theme	Phase 1 - Prepare	Phase 2 - Plan	Phase 3 - Experiment and Learn	Phase 4 - Sustain
**Strategy**	• Develop shared hospital vision and common strategic direction • Determine hospital’s improvement and change needs • Research and evaluate past lean implementations and identify potential adoption barriers in the organisation • Identify all stakeholders that will benefit from the value that lean might unlock	• Plan which adaptations should be made to adopt lean in the operating environment • Determine lean adoption strategy • Create a task specific adoption plan • Define stakeholder value and specify the criteria according to which the value that lean might realise will be assessed and measured against • Establish schedule for monthly lean performance meetings at top management level	• Top management to support lean adoption process • Contextualise lean for the specific hospital environment • Plan and create short-term wins • Document and monitor adaptations and learnings established during the adoption process • Measure the value that lean unlocks for all stakeholders according to planned criteria • Set lean performance meetings in place on a tactical and operational level	• Ensure organisational momentum by maintaining the strategy and common direction • Institutionalise lean goal setting across the entire hospital • Continuously measure the realised value of lean for all stakeholders and adapt strategy accordingly • Institutionalise lean performance meetings at strategic, tactical and operational level
**Resources**	• Assess which current supporting resources are available • Assess whether there is any current lean adoption and to what extent stakeholders have already been exposed to lean	• List and invest in supporting resources required • Obtain (external) experts who will provide employees with lean in healthcare training	• Put necessary supporting resources in place • Enlist external experts to co-guide adoption • Develop internal experts that will be involved in lean adoption and training of staff • Make process changes to align initial change vision with organisational processes	• Keep supporting resources such as technology up to date • Institutionalise change into current systems, SOPs and structures
**Engaging people**	• Obtain and ensure management commitment for lean adoption • Engage with all stakeholders and introduce the Lean philosophy • Create a sense of urgency emphasising change is necessary	• Identify lean champions from all levels of each organisational unit that can act as guiding change coalition • Appoint adoption team consisting of lean champions and other (front-line) employees • Specify supportive roles, processes and responsibilities of internal implementation team • Empower adoption team to lead the change by training them on lean principles, leadership and change management principles • Ensure the message that is conveyed about lean contributes to initial acceptance of lean and does not cause resistance to lean • Adopt organisational structure to ensure that hierarchies do not hinder teamwork to create value • Develop organisational performance feedback system • Communicate shared vision and common direction to all stakeholders	• Regularly evaluate performance of adoption team members related to lean adoption and commitment • Provide lean adoption feedback throughout the hospital • Communicate lean adoption progress to all stakeholders • Ensure performance feedback system triggers employee remedial action • Train all other staff on lean and empower them to identify waste in their respective processes • Establish inter-departmental cooperation • Gain acceptance of lean philosophy amongst all staff	• Provide continuous training and support to all staff
**Culture**	• Assess employee readiness for change • Assess hospital culture to determine whether the lean philosophy aligns with cultural philosophy	• Manage employee resistance to change and provide positive attention to those who embrace change • Establish supportive lean culture of continuous improvement • Identify and separate from past behaviour that is not conducive to a lean culture	• Reinforce lean culture of continuous improvement • Ensure management displays exemplary lean behaviour	• Normalise supportive lean culture of continuous improvement
**Change steps** ^**a**^	1: Assess the opportunity motivating the change 2: Select and support a guiding coalition 3: Formulate a clear compelling vision 5: Mobilise energy for change	2: Select and support a guiding coalition 4: Communicate the vision 5: Mobilise energy for change 6: Empower others to act 7: Develop and promote change-related knowledge and ability	2: Select and support a guiding coalition 4: Communicate the vision 5: Mobilise energy for change 6: Empower others to act 7: Develop and promote change-related knowledge and ability 8: Identify short-term wins and use as re-enforcement of the change process 9: Monitor and strengthen the change process	9: Monitor and strengthen the change process 10: Institutionalise the change in company culture and practices

^a^ Based on Stouten et al. [64]

### Phase 1: Prepare

As suggested by Zanon et al. [19], the first phase (Prepare) is associated with minor changes and setting the scene for lean implementation. In terms of the ‘strategy’ action items, following Grove et al. [37] and Lorden et al. [51] it is essential for a hospital to specify its (lean) strategic direction and improvement needs. It is key to contextualise how lean would fit into the hospital’s operating environment, the stakeholders of the lean adoption, and how they would benefit from lean adoption. Some stakeholders benefit more directly, such as patients, and others more indirectly such as suppliers. Furthermore, researching prior continuous improvement efforts and their successes and failures in a specific hospital is critical to setting the lean adoption strategy [14, 56]. These actions contribute to fulfilling Stouten et al.’s change step 1 [64].

‘Resources’ such as technology and trained lean staff members are required for a successful lean implementation in a hospital [52]. This implies the need to identify staff with previous exposure to lean in the form of training or practical lean experience. In addition, assessing whether other stakeholders are currently adopting lean is recommended to ensure alignment with their efforts and possibly leveraging from them. One must also identify technology currently in place that may ease team communication and enable aspects such as visual (performance) management in wards.

An initial engagement with people on lean and the value that may be realised will set the scene for the change initiative. In terms of ‘engaging people’, further involving management, staff members, and other stakeholders is characterised by change step 2 [64]. It is important to obtain management commitment for lean adoption at an early stage [51]. The underlying action items of this theme resonate with the ‘strategy’-related action items in that management needs to align the strategy of the organisation and hospital with the strategy of lean adoption. Moreover, communicating a sense of urgency to staff and introducing the lean philosophy will mobilise energy for change during the preparation phase.

During this initial engagement with employees, their readiness for change can be assessed [45]. A clear indication of employees’ change readiness is their realisation that the hospital needs process improvement [67]. Simultaneously the extent to which the hospital’s culture aligns with the lean philosophy will highlight behaviour that is not conducive to a lean culture. This will guide the implementation team in determining where to place their change efforts as the lean implementation progresses. Altogether, these action items allow an organisation to move on to the next phase.

### Phase 2: Plan

The planning phase is characterised by (initially) isolated lean improvements in the organisation [19]. The development of change-related knowledge and abilities is predominant in this phase [64]. With a clear company strategy in place from the preparation phase, the lean adoption strategy should be determined and set out in a clear adoption plan co-created by leaders at various hierarchical levels [3], for instance, by setting up monthly lean performance meetings at the top management level. Moreover, the specific value for various stakeholders anticipated by the lean adoption must be identified along with the criteria for measuring this value [74]. The value of lean in, for example, reducing waste such as waiting time that often occurs across all specialisations, can be articulated in this phase [1].

The planning phase provides the opportunity to list outstanding supporting resources and enlist external experts’ services to provide employees with the required lean knowledge and capability training specific to healthcare [17, 48] aligned with, change step 7 [64]. The engagement of people across the organisation is a priority during this phase [45]. This includes appointing a lean adoption team, ideally consisting of lean champions and other front-line staff. Since hospitals often have clear hierarchies in place that may limit teamwork [52], staff members from all organisational levels must be included as lean practitioners to curb any communication barriers. These employees must be informal leaders and have an inherent mindset of critical thinking and questioning the status quo [45]. This lean adoption team’s supportive roles, processes, and responsibilities must also be specified during this phase. The variability of patient demand often leads to the last-minute acute engagement of front-line staff in patient care and during scheduled lean activities. Hence, during the planning phase, the roles, processes and responsibilities in such scenarios must be clarified. Furthermore, the lean adoption team must be empowered to lead the lean change by providing them with training on lean, leadership, and change management principles. Altogether these change steps are clearly aligned with change steps 2, 6 and 7 [64].

As part of ‘engaging people’, the shared vision for lean and common direction that was determined during the preparation phase must now be communicated clearly (i.e., Stouten et al.’s [64] change step 4). Because this should lead to initial acceptance of lean (and not resistance), in the context of a fast-paced hospital environment, it should emphasise how value will be added and waste eliminated [75], allowing healthcare workers to focus on the quality of patient care.

Also measuring the progress of lean adoption will contribute to engaging people. Indeed, Noori [49] contends that quick wins are essential to motivate hospital staff towards lean adoption. Developing an organisational performance feedback system enables the measurement of the relationship between lean adoption and performance improvement across all levels of the organisation. The performance should be discussed at time intervals that align with strategic, tactical, and operational performance meetings. Bhasin [76] noted that such a lean performance management and measurement system needs to fit each organisational level to promote positive organisational behaviour and change acceptance. Possible performance indicators include reduced patient waiting time, improved resource utilisation, and patient satisfaction [76].

The measurement of lean adoption might also identify certain behaviours that are not conducive to a lean culture, leading to interventions to build a more supportive continuous improvement lean culture [45]. Once the change readiness of most employees has been determined and that the lean philosophy aligns with the cultural preferences of the hospital, the planning phase can be used to start establishing a supportive culture of continuous improvement and to manage resistance to change [52] by giving positive attention to those employees who embrace change.

### Phase 3: Experiment and learn

Each hospital has a unique operating environment and case mix [77]. A lean implementation maturity model must thus be contextualised as highlighted in the preparation phase. Therefore, the third phase has the longest duration, and this phase is associated with adapting lean according to the hospital’s specific requirements. This phase of lean maturity focuses on experimenting with lean adoption in various areas and proactively learning from this adoption by reviewing performance metrics.

From a strategic perspective, it is critical that top management support the lean adoption process and change its behaviour accordingly during this phase [50]. This may include revising some key performance indicators (KPIs) such as bed utilisation measures that management traditionally promotes [78]. Should such measurements prove to promote non-lean behaviour, top management needs to be proactive and change such KPIs. Installing lean performance meetings on a tactical and operational level will further assist in continuously learning from the lean adoption. These meetings provide a platform for discussing the measurement of lean’s value for stakeholders using the measurement criteria established in the planning phase [74]. Lastly, lean performance meetings will facilitate Stouten et al.’s [64] change step 4, 5, and 8. It is also beneficial to precisely plan and create short-term wins during this phase; those short-term successes can be used to reinforce the lean transformation (change step 8).

Change step 2 can be further executed by developing internal lean experts [52] through establishing a guiding coalition consisting of internal staff members. Although external experts enlisted during the previous phase may still be involved in the experiment and learn phase, their involvement will diminish over time as internal experts are developed [79] who could then start training other staff members during this phase. Using staff to train other employees on aspects of lean (such as the use of the unique lean vocabulary applicable to healthcare) can be a valuable tool in accelerating the adoption of lean while empowering staff to identify waste in their respective areas [35], which will mobilise the change and develop the required knowledge and abilities that promote the general acceptance of lean throughout the organisation (as described in Stouten et al.’s [64] change step 5 and 7). Inter-departmental cooperation is a hospital-specific aspect to establish for the realisation of this acceptance [36]. This tends to be a challenge, given the highly specialised disciplines in a hospital as well as the subsequent organisational silos that this creates [80].

Internal experts are referred to as lean champions, and the development of these resources is closely related to change step 6. Other supporting resources, such as software enabling knowledge management must also be provided [81]. Certain processes may need to be changed in such a way that they are aligned with the change vision set out in the preparation phase of the strategy theme. If, for example, the change vision of the hospital was set in the preparation phase to include collaboration between suppliers such as pathology and radiology services, process adaptations may include regular lean meetings between the front-line hospital staff and the supply staff.

The performance measurement system installed during the previous phase will act as a support tool through which the adoption team’s performance can regularly be evaluated. This action item also enables teams across units in the hospital to benchmark their performance in terms of achieving lean goals set out during the earlier phases, which will assist in eliminating organisational silos typical in hospitals whilst also embedding the lean adoption [70]. The feedback system must trigger remedial action so hospital staff can learn from mistakes [14] and make changes accordingly. Furthermore, feedback on lean adoption must be communicated throughout the hospital [51], providing all stakeholders with information on the implementation progress. From the above, it is clear that change steps 5, 6, 7 and 8 are addressed in the action items.

Most employees will experience changes in the hospital during the experiment and learn phase. It is important to reinforce the lean organisational culture of continuous improvement as the phase continues [53], whereby management needs to display exemplary lean behaviour [82]. Change steps 6 and 9 are clearly aligned with those actions. Finally, the experiment and learn phase is iterative in nature. As lean implementation and the associated action items of the SOLAR are progressing, it is important to modify actions to fit in with the specific hospital environment. For example, redefining the value that lean unlocks for some stakeholders may be necessary. This implies that some aspects of the planning phase should be amended.

### Phase 4: Sustain

The final phase of the SOLAR is characterised by the continuous monitoring of process improvements [19]. Change steps 9 and 10 promote the monitoring and institutionalisation of the change and are associated with this final phase [64]. Change will be institutionalised by maintaining the initial strategy and common direction [36]. It also remains important during this phase to continue to set lean goals and measure the value that lean realises for all stakeholders.

Resources such as technology and specific software need to be kept up-to-date, and changes to processes institutionalised by continuously updating standard operating procedures and staff structures [56]. Allocating resources to amend the reporting structure of certain units may be necessary. Furthermore, some KPIs, such as waiting time and its definition [1], may change as the hospital environment evolves. It remains critical to continue with lean training during this final phase whilst normalising the supportive lean culture of continuous improvement [48].

## Discussion

The high failure rate of continuous improvement initiatives [7] and lean implementation in hospitals [13] indicate a latent need for more clarity on how to adopt lean in a hospital setting. So far, however, there was no lean maturity model specific to healthcare or a hospital environment [19]. This is problematic because the hierarchical nature of healthcare is often a barrier to bottom-up improvement and the adoption of lean throughout the hospital (system-wide) instead of applying tools and techniques in isolation [11]. The SOLAR developed herein, therefore, responds to the need for a hospital-wide lean maturity model that takes into account the complexities of healthcare. In developing the SOLAR, relevant aspects from the literature were synthesised. As such, this research expands on the prolific lean implementation in healthcare literature by combining the known success factors with implementation science and change management theory. This makes the SOLAR simultaneously unique, comprehensive, and more practical.

A prominent complexity covered by the SOLAR is the primary goal of healthcare workers in hospitals to ensure the quality of patient care. Due to the sensitive nature of hospital settings and the human lives that are often at stake, hospital staff are more risk averse. A hospital is not the ideal setting for ‘trial and error’, often part of regular lean adoptions [70]. Continuous improvement initiatives may, therefore – initially – seem counter-intuitive for healthcare workers. However, since a key building block of the SOLAR is evidence-based implementation science, hospital staff are more likely to have confidence in using the SOLAR to guide them along their lean adoption journey. Furthermore, the SOLAR provides *guided* experimentation and learning in the third phase of sustainable lean implementation. The action items in this phase of the SOLAR allow hospitals to tailor implementation methods that are best suited to their unique operating context through guided experimentation and learning.

A second hospital-specific complexity is covered by integrating change management theory [64] throughout the SOLAR. Specialisation silos and hierarchies are often a barrier to sustainable lean implementation in hospitals [83]. The change coalition that is established in the planning phase of the SOLAR consists of staff from all units across all levels of the hospital which enables the permeation of barriers that existed because of hierarchies and silos. Consequently, the SOLAR emphasises the importance of identifying the stakeholders throughout the hospital that will be impacted by lean adoption in the preparation phase, as well as the subsequent defining of stakeholder value, and measuring and evaluating how lean adds value throughout the hospital. The concept of ‘value’, which is often conceptualised solely from the customer’s (the patient’s) perspective, is also determined for the different stakeholders. Ensuring that the perspectives of multiple stakeholders are proactively taken into account also reduces the risk of focusing on internal lean goals such as efficiency and cost reduction, which is often seen in public service settings [84].

In sum, in conjunction with the solid theoretical base, the SOLAR utilises input from lean healthcare practitioners and academics. As confirmed by them, the SOLAR is based on relevant theory and yet remains practical.

## Practical implications

The SOLAR was developed to be used by practitioners and academics as a practical guideline to test their approach on implementing lean in hospitals against. In particular, we envisage that top managers of hospitals, strategic advisors, and those in organisational development and continuous process improvement roles will find the SOLAR useful to tailor their hospital’s lean adoption approach. For example, using the SOLAR as an inspiration, data on the lean adoption progress can be captured by the lean adoption team and then discussed during periodical lean adoption strategy meetings. This data will be useful to (top) managers since it drives their learning process and informs decisions on support required to sustainably adopt lean.

The SOLAR also addresses the critical aspects pertaining to strategy, resources, the engagement of people, and organisational culture throughout lean adoption in hospitals. As mentioned, the SOLAR *guides* the implementation approach by emphasizing certain actions along the phases of the lean implementation journey in a hospital. This has proven to be a suitable characteristic of the SOLAR since respondents to the Delphi study agreed on its usefulness. Although the maturity phases of the SOLAR have been presented sequentially, there may be a need for hospitals implementing lean to revisit some of the phases as insights are gained during their lean implementation journey. Such tailoring to the unique hospital environment also enables hospital staff to accept lean adoption [83, 85]. While balancing between ‘theorising’ and ‘generalising’ as called for by Åhlström et al. [86], the model is further adaptable to local hospital environments.

## Limitations and future research

Creating an exhaustive list of items that need to be completed in a lean adoption journey is impossible, given that different contexts might require slightly different foci and organizational change is a dynamic process. And although we followed a systematic approach to reviewing the literature and Delphi study respondents have screened the SOLAR in various rounds, we may still have missed certain points. We propose that for purposive expert sampling, one could also consider approaching formal interest groups and associations with members who specialise in lean (i.e. the Lean Institute Africa, the Dutch Lean Healthcare community united in the ‘Lean in de Zorg’ (LIDZ) foundation, and the Lean Global Network).

Because some respondents in the Delphi study expressed the need for a more descriptive maturity model, this may be another valuable extension of our research. Reponen et al. [87] proposed a conceptual framework that can be used to benchmark lean performance in healthcare environments against best practices whilst taking the context of the environment into account. Since the inclusion of specific instructions on how to implement aspects such as training, communicating the strategy, and organising resources were not included in the aim of this research, the authors recommend that future research should include these aspects.

The next step is to validate the SOLAR in a hospital setting by further testing and possibly refining it. This can either be done retrospectively through a longitudinal study of hospitals that have implemented lean or as an intervention study following the action research approach [88, 89]. In the case of action research, positioning the SOLAR as a guideline for the lean implementation will be the starting point. Post-implementation focus groups can subsequently be used as a further validation tool of the SOLAR. We further propose to assess to what extent the lean intervention is brought about by the further operationalizing the action items of the SOLAR. One way to assess this is by using the PARTI (Participatory Action Research, Translation, and Implementation) model underpinned by implementation science [90].

## Conclusion

Hospitals are unique service environments that provide an essential and critical service to the community. Furthermore, hospitals tend to be high-pressure environments with variable demand and specialised services. These specialisations often result in silo structures which are hierarchical in nature and associated with waste and inefficiencies. Lean implementation in hospitals has, however, been proven to result in significant process improvements and enhanced quality of patient care. To address lean implementation efforts that are often not sustained in hospitals, we have developed the SOLAR: A unique maturity model that can act as a guideline for hospitals embarking on a lean implementation journey. After gathering expert feedback in three Delphi rounds, the SOLAR is suitable for use by academics and practitioners involved in lean deployment in hospitals, particularly because of its strong underpinning by implementation science and change management theory.

## Supplementary Information

Supplementary Material 1.  Delphi study survey questions

## Data Availability

The data used for the Systematic Literature Review was retrieved from publicly available internet databases as specified in the manuscript and is available from the authors upon reasonable request. The dataset for the Delphi study is not publicly available to protect the identity of respondents.

## Abbreviations

ENTREQ: Enhancing Transparency in Reporting the Synthesis of Qualitative Research
LIDZ: Lean in de Zorg
PARTI: Participatory Action Research, Translation, and Implementation
PRISMA: Preferred Reporting Items for Systematic reviews and Meta-Analyses
QIF: Quality Implementation Framework
SLR: Systematic Literature Review
SOLAR: Sustaining of Lean Adoption in Hospitals Roadmap

## References

[1] Van Zyl-Cillié M, Demirtas D, Hans E. Wait!What does that mean? Eliminating ambiguity of delays in healthcare from an OR/MS perspective. Health Syst. 2023;12(1):3–21. 10.1080/20476965.2021.2018362 PMC10013540 36926370

[2] Vanichchinchai A. Relationships among lean, service quality expectation and performance in hospitals. Int J Lean Six Sigma. 2022;13(2):457–73.

[3] Van Beers JCAM, Van Dun DH, Wilderom CPM. Effective hospital-wide lean implementation: top-down, bottom-up or through co-creative role modeling? Int J Lean Six Sigma. 2022;13(1):46–66.

[4] Van Rossum L, Aij KH, Simons FE, Van der Eng N, Ten Have WD. Lean healthcare from a change management perspective: the role of leadership and workforce flexibility in an operating theatre. J Health Organ Manag. 2016;30(3):475–93. 10.1108/JHOM-06-2014-0090 27119398

[5] Stelson P, Hille J, Eseonu C, Doolen T. What drives continuous improvement project success in healthcare? Int J Health Care Qual Assur. 2017;30(1):43–57. 10.1108/IJHCQA-03-2016-0035 28105876

[6] Akugizibwe AM, Clegg DR. Lean implementation: an evaluation from the implementers’ perspective. Int J Lean Enterp Res. 2014;1(2):132–61.

[7] Lameijer BA, Boer H, Antony J, Does RJMM. Continuous improvement implementation models: a reconciliation and holistic metamodel. Prod Plan Control. 2021;34(11):1062–81.

[8] Al-Balushi S, Sohal AS, Singh PJ, Hajri A, Al, Farsi YMA, Abri RA. Readiness factors for lean implementation in healthcare settings - a literature review. J Health Organ Manag. 2014;28:135–53. 10.1108/JHOM-04-2013-0083 25065107

[9] Stanković AK. Developing a lean consciousness for the clinical laboratory. J Med Biochem. 2008;27:354.

[10] Aherne J, Whelton J. Applying lean in healthcare. A collection of international case studies. J Chem Inform Modell. 2010. CRC Press.

[11] Lindsay CF, Aitken J. Using Programme Theory to evaluate lean interventions in healthcare. Prod Plan Control. 2024;35(8):824–41.

[12] Lameijer BA, De Mast J, Does R. Lean six Sigma deployment and maturity models: a critical review. Qual Manag J. 2017;24(4):6–20.

[13] Hallam CRA, Contreras C. Lean healthcare: scale, scope and sustainability. Int J Health Care Qual Assur. 2018;31(7):684–96. 10.1108/IJHCQA-02-2017-0023 30354875

[14] D’Andreamatteo A, Ianni L, Lega F, Sargiacomo M. Lean in healthcare: a comprehensive review. Health Policy. 2015;119:1197-209. 10.1016/j.healthpol.2015.02.002 25737260

[15] Henrique DB, Filho MG, Marodin G, Jabbour ABL, de Chiappetta Jabbour S. A framework to assess sustaining continuous improvement in lean healthcare. Int J Prod Res. 2021;59(10):2885–904.

[16] Kunnen YS, Roemeling OP, Smailhodzic E. What are barriers and facilitators in sustaining lean management in healthcare? A qualitative literature review. BMC Health Serv Res. 2023;23(1):958. 10.1186/s12913-023-09978-4 PMC10483794 37674182

[17] Andersen H, Røvik KA. Lost in translation: a case-study of the travel of lean thinking in a hospital. BMC Health Serv Res. 2015;15(1):1–9. 10.1186/s12913-015-1081-z PMC4578238 26390900

[18] Antony J, Sunder MV, Sreedharan R, Chakraborty A, Gunasekaran A. A systematic review of lean in healthcare: a global perspective. Int J Qual Reliab Manag. 2019;36:1370–91 Emerald Group Holdings Ltd.

[19] Zanon LG, Ulhoa TF, Esposto KF. Performance measurement and lean maturity: congruence for improvement. Prod Plan Control. 2021;32(9):760–74.

[20] Becker J, Knackstedt R, Pöppelbuß J. Developing maturity models for IT management. Bus Inf Syst Eng. 2009;1(3):213–22.

[21] Röglinger M, Pöppelbuß J. What makes a useful maturity model? a framework of general design principles for maturity models and its demonstration in business process management. 2011.

[22] Maier AM, Moultrie J, Clarkson PJ. Assessing organisational capabilities: reviewing and guiding the development of maturity grids. IEEE Trans Eng Manag. 2012;59(1):138–59.

[23] Marsilio M, Pisarra M, Rubio K, Shortell S. Lean adoption, implementation, and outcomes in public hospitals: benchmarking the US and Italy health systems. BMC Health Serv Res. 2022;22(1):122. 10.1186/s12913-022-07473-w PMC8800363 35090455

[24] do Mourão CAM, de Miranda Filho AN, Nogueira RN, de Barros Neto JP, da Costa JM. Using storytelling to understand a company’s lean journey. In: IGLC 2021–29th Annual Conference of the International Group for Lean Construction - Lean Construction in Crisis Times: Responding to the Post-Pandemic AEC Industry Challenges. Department of Engineering, Civil Engineering Division, Pontificia Universidad Catolica del Peru; 2021: 423–32.

[25] Jayaraman R. Lean management maturity in the Indian industry: results from a longitudinal exploratory study using exploratory factor analysis. Int J Oper Quant Manag. 2021;27(2):81–110.

[26] Jami Pour M, Jafari SM. Toward a maturity model for the application of social media in healthcare: the health 2.0 roadmap. Online Inf Rev. 2019;43(3):404–25.

[27] Tortorella GL, Vergara LGL, Ferreira EP. Lean manufacturing implementation: an assessment method with regard to socio-technical and ergonomics practices adoption. Int J Adv Manuf Technol. 2017;89(9–12):3407–18.

[28] Jørgensen F, Matthiesen R, Nielsen J, Johansen J. Lean Maturity, Lean Sustainability. In: Advances in Production Management Systems: International IFIP TC 5, WG 57 Conference on Advances in Production Management Systems. Linkoping: Springer; 2007: 371–8.

[29] Tortorella GL, Marodin GA, Fogliatto FS, Miorando R. Learning organisation and human resources management practices: an exploratory research in medium-sized enterprises undergoing a lean implementation. Int J Prod Res. 2015;53(13):3989–4000.

[30] Tortorella GL, Fogliatto FS. Method for assessing human resources management practices and organisational learning factors in a company under lean manufacturing implementation. Int J Prod Res. 2014;52(15):4623–45.

[31] Verrier B, Rose B, Caillaud E. Sustainable manufacturing through lean and green approach: best practices and indicators. In: Volume 2A: 33rd Computers and Information in Engineering Conference. American Society of Mechanical Engineers; 2013.

[32] Mettler T, Rohner P. Situational maturity models as instrumental artifacts for organisational design. In: Proceedings of the 4th International Conference on Design Science Research in Information Systems and Technology, DESRIST ’09. 2009.

[33] Netland TH, Ferdows K. The S-curve effect of lean implementation. Prod Oper Manag. 2016;25(6):1106–20.

[34] Negrão LLL, Lopes de Sousa Jabbour AB, Latan H, Godinho Filho M, Chiappetta Jabbour CJ, Ganga GMD. Lean manufacturing and business performance: testing the S-curve theory. Prod Plan Control. 2020;31(10):771–85.

[35] Aij KH, Simons FE, Widdershoven GAM, Visse M. Experiences of leaders in the implementation of lean in a teaching hospital - barriers and facilitators in clinical practices: a qualitative study. BMJ Open. 2013;3(10):e003605. 10.1136/bmjopen-2013-003605 PMC3816237 24171938

[36] de Souza LB, Pidd M. Exploring the barriers to lean health care implementation. Public Money Manag. 2011;31(1):59–66.

[37] Grove AL, Meredith JO, Macintyre M, Angelis J, Neailey K. UK health visiting: challenges faced during lean implementation. Leadersh Health Serv. 2010;23(3):204–18.

[38] Mazur LM, Rothenberg L, McCreery JK. Measuring and understanding change recipients’ buy-in during lean program implementation efforts. In: 61st Annual IIE Conference and Expo Proceedings. 2011.

[39] Sobek IIDK. Lean healthcare implementation: critical success factors. 61st Annual IIE Conference and Expo Proceedings. 2011.

[40] Steed A. An exploration of the leadership attributes and methods associated with successful lean system deployments in acute care hospitals. Qual Manag Health Care. 2012;21(1):48–58. 10.1097/QMH.0b013e318241825c 22207019

[41] Maijala R, Eloranta S, Reunanen T, Ikonen TS. Successful implementation of lean as managerial principle in health care: a conceptual analysis from systematic literature review. Int J Technol Assess Health Care. 2018;34(2):134–46. 29642955 10.1017/S0266462318000193

[42] Page MJ, McKenzie JE, Bossuyt PM, Boutron I, Hoffmann TC, Mulrow CD, Shamseer L, Tetzlaff JM, Akl EA, Brennan SE, Chou R. The PRISMA 2020 statement: an updated guideline for reporting systematic reviews. BMJ. 2021;372:n71. 10.1136/bmj.n71 PMC8005924 33782057

[43] Siddaway AP, Wood AM, Hedges LV. How to do a systematic review: a best practice guide for conducting and reporting narrative reviews, meta-analyses, and meta-syntheses. Annu Rev Psychol. 2019;70(1):747–70. 10.1146/annurev-psych-010418-102803 30089228

[44] Tong A, Flemming K, McInnes E, Olivier S, Craig J. Enhancing transparency in reporting the synthesis of qualitative research: ENTREQ. BMC Med Res Methodol. 2012;12:1–8. 23185978 10.1186/1471-2288-12-181 PMC3552766

[45] Jain V, Ajmera P. Modelling of the factors affecting lean implementation in healthcare using structural equation modelling. Int J Syst Assur Eng anag. 2019;10(4):563–75.

[46] Noori B. Identifying critical issues in lean implementation in hospitals. Hosp Top. 2015;93(2):44–52. 26185933 10.1080/00185868.2015.1052299

[47] Ajmera P, Jain V. A fuzzy interpretive structural modeling approach for evaluating the factors affecting lean implementation in Indian healthcare industry. Int J Lean Six Sigma. 2020;11(2):376–597.

[48] Régis TKO, Gohr CF, Santos LC. Lean healthcare implementation: experiences and lessons learned from Brazilian hospitals. RAE Revista De Administracao De Empresas. 2018;58(1):30–43.

[49] Noori B. The critical success factors for successful lean implementation in hospitals. Intl J Prod Qual Manag. 2015;15(1):108–26.

[50] Patri R, Suresh M. Factors influencing lean implementation in healthcare organisations: an ISM approach. Int J Health Manag. 2018;11(1):25–37.

[51] Lorden AL, Zhang Y, Lin SH, Côté MJ. Measures of success: the role of human factors in lean implementation in healthcare. Qual Manag J. 2014;21(3):26–37.

[52] Harrison MI, Paez K, Carman KL, Stephens J, Smeeding L, Devers KJ, et al. Effects of organisational context on lean implementation in five hospital systems. Health Care Manage Rev. 2016;41(2):127–44. 25539057 10.1097/HMR.0000000000000049

[53] Ulhassan W, Sandahl C, Westerlund H, Henriksson P, Bennermo M, Von Thiele Schwarz U et al. Antecedents and characteristics of lean thinking implementation in a Swedish hospital: a case study. Qual Manag Health Care. 2013;22(1):48–61. 10.1097/QMH.0b013e31827dec5a 23271593

[54] Poksinska B. The current state of lean implementation in health care: literature review. Qual Manag Health Care. 2010;19(4):319–29. 10.1097/QMH.0b013e3181fa07bb 20924253

[55] Nilsen P. Making sense of implementation theories, models and frameworks. Implement Sci. 2015;10(1):53–79. 10.1186/s13012-015-0242-0 PMC4406164 25895742

[56] Holden RJ. Lean thinking in emergency departments: a critical review. Ann Emerg Med. 2011;57(3):265–73. 10.1016/j.annemergmed.2010.08.001 PMC6548198 21035904

[57] Eccles MP, Mittman BS. Welcome to implementation science. Implement Sci. 2006;1:1.

[58] May C, Finch T. Implementing, embedding, and integrating practices: an outline of normalisation process theory. Sociology. 2009;43(3):535–54.

[59] Tabak RG, Khoong EC, Chambers DA, Brownson RC. Bridging research and practice: models for dissemination and implementation research. Am J Prev Med. 2012;43:337–50. 10.1016/j.amepre.2012.05.024 PMC3592983 22898128

[60] Greenhalgh T, Robert G, Macfarlane F, Bate P, Kyriakidou O. Diffusion of innovations in service organisations: systematic review and recommendations. Milbank Q. 2004;82(4):581–629. 15595944 10.1111/j.0887-378X.2004.00325.x PMC2690184

[61] Meyers DC, Durlak JA, Wandersman A. The quality implementation framework: a synthesis of critical steps in the implementation process. Am J Community Psychol. 2012;50(3–4):462–80. 10.1007/s10464-012-9522-x 22644083

[62] Al-Haddad S, Kotnour T. Integrating the organisational change literature: a model for successful change. J Organ Chang Manag. 2015;28(2):234–62.

[63] Harrison R, Fischer S, Walpola RL, Chauhan A, Babalola T, Mears S, et al. Where do models for change management, improvement and implementation meet? A systematic review of the applications of change management models in healthcare. J Healthc Leadersh. 2021;13:85–108 Dove Medical Press Ltd. 33737854 10.2147/JHL.S289176 PMC7966357

[64] Stouten J, Rousseau DM, De Cremer D. Successful organisational change: integrating the management practice and scholarly literature. Acad Manag Annals. 2018;12(2):752–88.

[65] Langley A, Smallman C, Tsoukas H, Van De Ven AH. Process studies of change in organisation and management: unveiling temporality, activity, and flow. Acad Manag J. 2013;56(1):1069–70.

[66] Rousseau DM, ten Have S. Evidence-based change management. Organ Dyn. 2022;51(3):100899.

[67] Errida A, Lotfi B. The determinants of organisational change management success: literature review and case study. Int J Eng Bus Manag. 2021;13:1847970211016273.

[68] Martin N, Fischer DA, Kerpedzhiev GD, Goel K, Leemans SJJ, Röglinger M, et al. Opportunities and challenges for process mining in organisations: results of a Delphi study. Bus Inf Syst Eng. 2021;63(5):511–27.

[69] Skinner R, Nelson RR, Chin WW, Land L. The Delphi method research strategy in studies of information systems. Commun Association Inform Syst. 2015;37:31–63.

[70] Prado-Prado JC, Fernández-González AJ, Mosteiro-Añón M, García-Arca J. Increasing competitiveness through the implementation of lean management in healthcare. Int J Environ Res Public Health. 2020;17(14):1–26. 10.3390/ijerph17144981 PMC7400224 32664355

[71] Skulmoski GJ, Hartman FT, Krahn J. The Delphi method for graduate research. J Inf Technol Educ Res. 2007;6(1):1–21.

[72] Adler M, Ziglio E. Gazing into the oracle: The Delphi method and its application to social policy and public health. Jessica Kingsley; 1996.

[73] Trochim W, Donnelly JP. The research methods knowledge base. 3rd ed. Thompson Publishing Group; 2007.

[74] Franken JC, van Dun DH, Wilderom CP. Kaizen event process factors for operational performance improvement: an archival study. Prod Plan Control 2024:1–5.

[75] Bakke AL, Johansen A. Implementing of lean - challenges and lessons learned. Procedia Computer Science. Elsevier B.V.; 2019. pp. 373–80.

[76] Bhasin S. Lean and performance measurement. J Manuf Technol Manag. 2008;19(5):670–84.

[77] Hulshof PJH, Kortbeek N, Boucherie RJ, Hans EW, Bakker PJM. Taxonomic classification of planning decisions in health care: a structured review of the state of the art in OR/MS. Health Syst. 2012;1(2):129–75.

[78] Khalifa M, Khalid P. Developing strategic health care key performance indicators: a case study on a tertiary care hospital. Procedia Computer Science. Elsevier B.V.; 2015. pp. 459–66.

[79] Holmemo MDQ, Powell DJ, Ingvaldsen JA. Making it stick on borrowed time: the role of internal consultants in public sector lean transformations. TQM J. 2018;30(3):217–31.

[80] Drupsteen J, Van der Vaart T, Van Donk DP. Integrative practices in hospitals and their impact on patient flow. Int J Oper Prod Manag. 2013;33(7):912–33.

[81] Zhang B, Niu Z, Liu C. Lean tools, knowledge management, and lean sustainability: the moderating effects of study conventions. Sustain (Switz). 2020;12(3):956.

[82] Tortorella G, Van Dun DH, De Almeida AG. Leadership behaviors during lean healthcare implementation: a review and longitudinal study. J Manuf Technol Manage. 2020;31(1):193–215.

[83] Rosa A, Marolla G, Lega F, Manfredi F. Lean adoption in hospitals: the role of contextual factors and introduction strategy. BMC Health Serv Res. 2021;21(1):1–8. 10.1186/s12913-021-06885-4 PMC8403367 34454500

[84] Radnor Z, Johnston R. Lean in UK Government: internal efficiency or customer service. Prod Plan Control. 2013;24(10–11):903–15.

[85] Hung D, Gray C, Martinez M, Schmittdiel J, Harrison MI. Acceptance of lean redesigns in primary care. Health Care Manage Rev. 2017;42(3):203–12. 26939032 10.1097/HMR.0000000000000106

[86] Åhlström P, Danese P, Hines P, Netland TH, Powell D, Shah R, et al. Is lean a theory? Viewpoints and outlook. Int J Oper Prod Manag. 2021;41(12):1852–78.

[87] Reponen E, Rundall TG, Shortell SM, Blodgett JC, Juarez A, Jokela R et al. Benchmarking outcomes on multiple contextual levels in lean healthcare: a systematic review, development of a conceptual framework, and a research agenda. BMC Health Serv Res. 2021;21(1):1–18. 10.1186/s12913-021-06160-6 PMC7893761 33607988

[88] Coughlan P, Coghlan D. Action research for operations management. Int J Oper Prod Manag. 2002;22(2):220–40.

[89] Oliva R. Intervention as a research strategy. J Oper Manag. 2019;65(7):710–24.

[90] Fitzgerald A, Hayes K, Curry J, Eljiz K, Radford K. Balancing Yin and Yang: the development of a framework using participatory action research for the translation and implementation (part 1) of new practices. Asia Pac J Health Manag. 2016;11:14–24.

