# Supplementary material for: Toward a roadmap for sustainable lean adoption in hospitals: a Delphi study
Source: BMC Health Serv Res. 2024 Sep 18;24:1088. doi: 10.1186/s12913-024-11529-4 (PMC11409581; doi:10.1186/s12913-024-11529-4)
Supplement: Supplementary file 1 — Supplementary Material 1. Delphi study survey questions [file 12913_2024_11529_MOESM1_ESM.docx]

Additional File 1:

Toward a Roadmap for Sustainable Lean Hospital Adoption:

A Delphi Study

This additional file shows the survey questions developed for each Delphi round in our data collection.

1. **Delphi round 1**

*Table S1: Survey questions Delphi round 1*

| 1. Please indicate whether you agree with the following statements concerning the problems addressed in this study | | | | | | | |
| --- | --- | --- | --- | --- | --- | --- | --- |
| Lean has the capability to increase a hospital’s quality of  service and performance | Strongly Agree | Agree | | Undecided | Disagree | | Strongly Disagree |
| Although initial lean implementations might be successful it is  not sustained in the long run | Strongly Agree | Agree | | Undecided | Disagree | | Strongly Disagree |
| 1. Please indicate whether you agree with the following statements concerning the SLIR. | | | | | | | |
| The SLIR contributes towards the sustainability of lean  implementations in a hospital environment | Strongly Agree | Agree | | Undecided | Disagree | | Strongly Disagree |
| The 7 dimensions of the SLIR represents critical areas where  maturity needs to be achieved in order for lean  implementations to be sustained | Strongly Agree | Agree | | Undecided | Disagree | | Strongly Disagree |
| 1. Please indicate whether you agree that the SLIR adheres to the respective design requirements. | | | | | | | |
| The model is applicable to any hospital environment with the necessary contextualisation | Strongly Agree | Agree | | Undecided | Disagree | | Strongly Disagree |
| The model facilitates sustainable lean implementation | Strongly Agree | Agree | | Undecided | Disagree | | Strongly Disagree |
| Staff members from all (relevant) levels will be able to interpret and apply the model | Strongly Agree | Agree | | Undecided | Disagree | | Strongly Disagree |
| Staff members from various (relevant) disciplines will be able to interpret and apply the model | Strongly Agree | Agree | | Undecided | Disagree | | Strongly Disagree |
| Maturity and the steps to achieve maturity within maturity levels is clearly defined | Strongly Agree | Agree | | Undecided | Disagree | | Strongly Disagree |
| Maturity levels from a logical maturation path toward sustainable lean implementation | Strongly Agree | Agree | | Undecided | Disagree | | Strongly Disagree |
| 1. How many years of experience on lean implementation in a healthcare environment do you have? | | | | | | | |
| 1. Please indicate in which area(s) lies your experience within lean in healthcare implementation | Academic | | Private  Healthcare | | | Public Healthcare | |
| 1. Please provide any additional comments | | | | | | | |

1. **Delphi round 2**

*Table S2: Survey questions Delphi round 2*

| 1. The following section deals with your opinion about lean implementation in hospitals. Please indicate whether you agree with the following statements concerning the problem addressed in this study: | | | | | | | | | | | | | | |
| --- | --- | --- | --- | --- | --- | --- | --- | --- | --- | --- | --- | --- | --- | --- |
| Lean implementation in hospitals has increased over the past two decades | Strongly Agree | Agree | | | | | Undecided | | | | Disagree | | | Strongly Disagree |
| Lean has the capability to increase a hospital’s quality of service and performance | Strongly Agree | Agree | | | | Undecided | | | | Disagree | | | | Strongly Disagree |
| Although initial lean implementations in hospitals might be successful it is not sustained in the long run |  |  | | | |  | | | |  | | | |  |
| 1. Before you continue with the next part of the survey, please take a more detailed look at the SLIR model pdf document sent to you in our email.This section asks your opinion on the use of the SLIR model | | | | | | | | | | | | | | |
| The SLIR contributes towards the sustainability of lean implementations in a hospital | Strongly Agree | Agree | | | | Undecided | | | Disagree | | | | | Strongly Disagree |
| The four themes of Strategy, Resources, Engaging People and Culture that are on the x-axis of the SLIR along with their action items displayed in the SLIR represent critical areas where maturity needs to be achieved in order for lean implementations to be sustained | Strongly Agree | Agree | | | | Undecided | | | Disagree | | | | | Strongly Disagree |
| Once the four maturity phases of Prepare, Plan, Experiment & Learn and Sustain on the y-axis of the SLIR are completed, Lean implementation in a hospital will be sustained over the long term |  |  | | | |  | | |  | | | | |  |
| The change steps on the z-axis of the SLIR clearly guides the user of the SLIR on which change management steps needs to be covered to successfully implement and sustain Lean in a hospital |  |  | | | |  | | |  | | | | |  |
| 1. This section aims to assess the ease of use of the SLIR | | | | | | | | | | | | | | |
| The model is applicable to any hospital | Strongly Agree | | Agree | | Undecided | | | | | | | Disagree | | Strongly Disagree |
| The action items of the SLIR address all the relevant steps that need to be taken to successfully implement and sustain Lean in a hospital | Strongly Agree | | Agree | | Undecided | | | | | | | Disagree | | Strongly Disagree |
| Staff members from all (relevant) levels will be able to interpret and apply the model | Strongly Agree | | Agree | | Undecided | | | | | | | Disagree | | Strongly Disagree |
| 1. This section enables you to elaborate on your feedback. Please provide any final suggestions you would have to improve the SLIR in the space provided below. | | | | | | | | | | | | | | |
| 1. If you have any other comments you would like to share with the researcher, please do so in the space provided below | | | | | | | | | | | | | | |
| 1. How many years of experience on lean implementation in a healthcare environment do you have? | | | | | | | | | | | | | | |
| 1. Please indicate in which area(s) your experience of lean in healthcare lies | Public Healthcare | | | Private Healthcare | | | | Academic Institution | | | | | Other | |

1. **Delphi round 3**

*Table S3: Narrative discussion questions Delphi round 3*

| 1. Do you agree on the renaming of the maturity model? |
| --- |
| 1. Is the presentation of the phases of the model more clear? |
| 1. What are your thoughts on stakeholder identification and co-creation of leaders? |
| 1. Do we address your specific feedback and suggestions? |
